# Phα1β interaction with the Kv11.1 potassium channel in HEK293 cells transfected with the human ERG channel

**DOI:** 10.1590/1678-9199-JVATITD-2024-0039

**Published:** 2025-01-20

**Authors:** João B. Calixto, Adara Aurea dos Santos, Juliano Ferreira, Alessandra Hubner Souza, Célio José de Castro, Marcus Vinicius Gomez

**Affiliations:** 1 Center for Innovation and Pre-Clinical Trials (CIEnP), Florianópolis, SC, Brazil.; 2 Federal University of Santa Catarina (UFSC), Florianópolis, SC, Brazil.; 3 School of Medical Sciences of Minas Gerais (FCM-MG), Belo Horizonte, MG, Brazil.; 4 School of Health Santa Casa BH, Belo Horizonte, MG, Brazil.

**Keywords:** Phα1β, analgesic, Dofetilide, Kv11.1 potassium channel, hERG channel interaction, HEK293-hERG, cell viability

## Abstract

**Background:**

This study examines the impact of Phα1β, a spider peptide derived from the venom of *Phoneutria nigriventer*, on the Kv11.1 potassium channel in HEK293 cells transfected with the human ERG potassium channel. Phα1β inhibits high-voltage calcium channels and acts as an antagonist of the TRPA1 receptor, both of which play crucial roles in pain transduction pathways. Over the past 15 years, our research has demonstrated the potential of Phα1β, in both its native and recombinant forms, as a promising analgesic drug through preclinical tests conducted on rodent pain models. Regulatory agencies require the evaluation of new drugs on human ERG channels.

**Methods:**

To assess hERG potassium channel inhibition, we utilized the FLIPR® Potassium Assay, a commercially available kit. The assay involved testing the effects of Phα1β alongside the well-established hERG potassium channel blocker dofetilide, which served as a positive control. The viability of HEK-293 cells was assessed using the colorimetric MTT reduction test (3-(4, dimethylthiazol-2-yl)-2,5-diphenyltetrazolium bromide), whereby viable cells reduce the MTT salt, forming a formazan complex within their mitochondria, as previously described.

**Results:**

Phα1β was tested at concentrations of 56, 225, 450, and 900 pMol, resulting in a discreet inhibition of hERG potassium channel activity at higher concentrations, approximately 13.47%, with an IC_50_ value exceeding 900 pMol. Dofetilide, administered at concentrations ranging from 0.0001 to 10 µM, displayed a concentration-dependent inhibition of the hERG potassium channel, with a mean IC_50_ value of 0.1642 µM (0.1189-0.2282 µM). To evaluate cytotoxicity, HEK293-hERG cells were exposed to Phα1β concentrations of 56/900 pMol for 24 hours, resulting in no significant alteration in cell viability.

**Conclusion:**

Our findings indicate that even at high concentrations, Phα1β does not impede the functionality of the hERG potassium channel nor affect cell viability.

## Background

In the last two decades, considerable research has focused on N-type calcium channel inhibitors to develop novel analgesic drugs [1]. The ω-conotoxin MVIIA, derived from the snail *Conus magnus,* underwent synthesis into a compound known as ziconotide, which is commercially available under the name Prialt®. Ziconotide is a selective, reversible, and potent blocker of N-type high-voltage-sensitive calcium channels and is an effective agent for pain control [2]. However, the drug produces maximal analgesia at doses close to its toxic threshold, causing severe side effects. Ziconotide was developed as a first-class analgesic drug for neuropathic pain. Yet, its narrow therapeutic window and adverse effects limit its clinical use in patients [3-4]. Pharmacological management of severe chronic pain remains challenging with currently available analgesic drugs, highlighting a significant unmet therapeutic need. Currently, neuropathic pain management is unsatisfactory and remains a challenge in clinical practice [4], and the search for new effective and safe analgesic drugs is necessary. Phα1β, a spider peptide purified from the venom of *Phoneutria nigriventer* [5], demonstrates analgesic effects through inhibitory actions on high-voltage calcium channels [6], with a specific preference for N-type channels. Additionally, it acts as an antagonist of the TRPA1 receptor, a significant pathway involved in pain transduction [7]. The dual activity of Phα1β in analgesia suggests a potential advantage and could broaden its efficacy in various pain-related conditions [8, 9].

We have compared the analgesic effects and side effects of native and recombinant Phα1β with ziconotide, administered intrathecally, in several rodent models of pain, including neuropathic pain [10]. The results indicated that both native and recombinant Phα1β exhibited a higher analgesic profile than ziconotide, and more importantly, its analgesic properties were associated with fewer side effects [11]. Some advantages of the antinociceptive action of Phα1β over ziconotide, a first-class analgesic drug, were observed. The IC_50_ (50% of the inhibitory dose) for Phα1β on release of the excitatory glutamate induced by capsaicin in nerve endings is 2.1 µM, three times lower than the IC_50_ for ziconotide, which is 6.2 µM [12]. The therapeutic window of Phα1β is 16, while that of ziconotide is 4 [13]. Ziconotide is 2.7 times more toxic than Phα1β. The DT_50_ (50% of the toxic dose) for Phα1β is 788 pMol, while for ziconotide, it is 287 pMol, which is very close to its effective action [13], limiting its clinical use [4]. Phα1β improved neuroinflammatory responses in a multiple sclerosis mouse model with higher efficacy than ziconotide [14, 15]. Phα1β induces analgesic effects in a model of cancer pain [16, 17].

Astrocyte proliferation is a pathological hallmark of peripheral inflammation, which can be reversed by Phα1β treatment, while ziconotide has no effect [18]. In rats, intravenous administration of ω-conotoxin MVIIA decreased blood pressure, while recombinant intravenous Phα1β induced analgesia in neuropathic pain with negligible cardiac problems [19]. In the treatment of cerulein-induced pancreatitis in rats, ω-conotoxin MVIIA, contrary to the effect of Phα1β, did not affect the cerulein-induced increase in serum amylase and lipase levels [20]. Phα1β relieves nociception induced by nerve deafferentation in rats [21], and the recombinant form presents a good safety profile with transient toxicity in clinical signals at doses higher than those used to achieve analgesic effect [22]. Phα1β reverses morphine tolerance and enhances the analgesic effect of morphine, providing preclinical evidence of Phα1β as an adjuvant drug in opioid treatment [23]. The above comparisons between the analgesic actions of ω-conotoxin MVIIA and Phα1β suggest that Phα1β has the potential to become a new analgesic drug. 

Regulatory agencies, such as the Food and Drug Administration (FDA) and the European Medicines Agency, which oversee the registry of new drugs, have several requirements for the approval of a new drug. The FDA has issued guidelines (ICH S6 and S7A) for the evaluation of novel chemical drugs during development to assess their potential to induce QT prolongation. QT prolongation is an indicator of a serious adverse effect that can cause ventricular arrhythmia, potentially leading to sudden death [24]. Cardiotoxicity is a frequent problem observed during the early stages of drug discovery and development, often leading to the early discontinuation of promising candidates. It has been estimated that nearly 70% of new drugs tested are eliminated at early stages, primarily due to ERG-related safety issues [25, 26, 27], thereby limiting the number of drugs that enter the development pipeline [28]. The recombinant HEK293 line expressing the human ERG potassium channel - also known as KCNH2 or Kv11.1 from BPS Bioscience (accession number NM_000238, growth medium 1B BPS #79531, and thaw medium 1 #60187) - was used in this study.

The present study describes the evaluation of the recombinant Phα1β interaction with the KV11.1 potassium channel in HEK293 cells transfected with the human ERG potassium channel [29, 30].

## Methods

### Recombinant Phα1β

Giotto Biotech^®^ (https://www.giottobiotech.com) synthesized the recombinant version of Phα1β via *Escherichia coli* expression for evaluation. It was purified through a proprietary production process that combined ion exchange and size exclusion chromatography. The yield of the process was 0.5 mg/mL. The peptide molecular weight (Mw) was 6,045 kDa. Both native and the recombinant Phα1β share the same 55 amino acid sequence: 

ACIPRGEICTDDCECCGCDNQCYCPPGSSLGIFKCSCAHANKYFCNRKKEKCKKA

The sequence of the amino acids in the recombinant and natural Phα1β peptide is identical, except for the addition of a methionine at the N-terminal portion of the recombinant peptide (the addition of the starting methionine is a common practice in heterologous protein expression). The purity of the recombinant toxin is higher than 90%, as demonstrated in an SDS-PAGE assay. 

### Evaluation of Phα1β and dofetilide interactions on the Kv11.1 potassium channel in HEK293 cells transfected with the human ERG potassium channel

The recombinant EK293 cell line expressing the human ERG potassium channel - also known as KCNH2 or Kv11.1 from BPS Bioscience (accession number NM_000238, growth medium 1B BPS #79531, and thaw medium 1 #60187) - was used in this study. Cells were thawed and cultured according to the supplier's specifications: hERG (Kv11.1)-HEK-293 Recombinant Cell Line #60619 was used as described. Cells transfected with the human hERG potassium channel were subcultured in 96-well plates and maintained under controlled conditions for 24 hours. They were then incubated with the probe for 1 hour before adding the 30-minute treatments, followed by reading on the FlexStation. 

The interaction of recombinant Phα1β and dofetilide with the hERG potassium channel was assessed using the commercial kit FLIPR Potassium Assay. The assay was performed according to the manufacturer's specifications. The FLIPR^®^ Potassium Assay kit contains a thallium-sensitive indicator dye. During the initial dye-loading step, thallium, in the form of the acetoxymethyl ester (AM), enters the cells by passive diffusion across the cell membrane. Cytoplasmic esterases cleave the AM ester, releasing its active fluorogenic form. A masking dye is applied extracellularly to reduce background fluorescence. To activate the potassium channel, the cells are stimulated with either a mixture of K+ and TI+ or a ligand in the presence of TI+. The increase in fluorescent signal represents the influx of TI+ into the cell, specifically through potassium channels. This increase in signal functionally measures potassium channel activity. For the assay, cells were plated in 96-well plates (5 × 10^4^ cells/well) of the HEK-293 cell line (BPS Bioscience), expressing the human ERG potassium channel. After 24 hours, the culture medium was aspirated and replaced with 50 µL of calcium- and magnesium-free HBSS. The cells were then incubated with 50 µL of the fluorescent probe present in the commercial kit, containing probenecid (Sigma-Aldrich) at a final concentration of 2.5 mM per well. After 1 hour of incubation at room temperature and in the absence of light, 25 µL of Phα1β at concentrations of 56, 225, 450, and 900 pMol or dofetilide (Sigma-Aldrich), an hERG potassium channel inhibitor, at concentrations of 0.0001 to 10 µM were incubated with the cells for 30 minutes. The stimulus buffer was added to each column through automated pipetting present in the FlexStation 3 equipment. Data were obtained using the SoftMax^®^ Pro Software at a wavelength of 485/525 nm. Data analysis was performed using SoftMax Pro Software and GraphPad Prism^®^. 

### Influence of Phα1β on HEK-293 cell viability

The viability assay of HEK-293 cells was performed using the colorimetric MTT reduction test (3-(4, dimethylthiazol-2-yl)-2,5-diphenyltetrazolium bromide), whereby viable cells reduce the MTT salt, forming a formazan complex within their mitochondria, as previously described. For this assay, after treating HEK-293 cells (5 × 10^4^ cells/well) with Phα1β (56-900 pMol), the culture medium was replaced with medium containing MTT (0.5 mg/mL), and the cells were incubated for approximately 1 hour and 30 minutes at 37 ºC ± 0.1 °C in a humidified atmosphere containing 5% ± 0.1% CO_2_. Subsequently, the MTT solution was removed, and 200 µL of dimethyl sulfoxide was added. Absorbance was measured at 570 nm and 485 nm using the spectrophotometer (SpectraMax MiniMax 300, Imaging Cytometer, Molecular Device). Data analysis was performed using SoftMax Pro Software and GraphPad Prism^®^. Statistical analysis was conducted using GraphPad Prism^®^ software (GraphPad Software Inc., San Diego, CA, USA). The results were expressed as SD ± 0.02 average standard. Absorbance was measured at 570 nm using the spectrophotometer (SpectraMax MiniMax 300, Imaging Cytometer, Molecular Device). Data analysis was performed using SoftMax Pro Software and GraphPad Prism^®^.

## Results

### Interaction of dofetilide and Phα1β with Kv11.1 potassium channels

**Figure 1.**
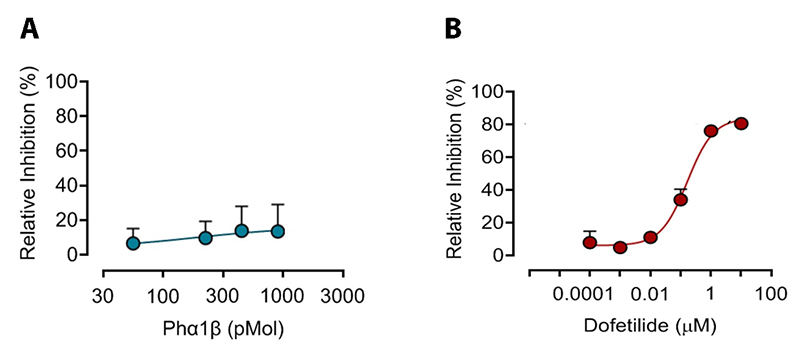
Concentration-response curves for the blockade of hERG channel current by **(A)** Phα1β and **(B)** dofetilide as assessed using a FLIPR^®^ potassium assay. HEK293 cells transfected with hERG were incubated with Phα1β (56-900 pM) or dofetilide (0.0001-10 μM) for 30 minutes, followed by the addition of 1 mM thallium + 10 mM potassium from an automated FlexStation 3, which was also used to monitor changes in fluorescence (excitation 485 nm, emission 538 nm) with subsequent analysis using SoftMax Pro 7.1 software. The y-axis indicates the percentage of inhibition relative to normal channel activity seen in the absence of the antagonist. The IC_50_ values for Phα1β and dofetilide were > 900 pM and 0.1642 μM, respectively. The points represent the mean ± SD of three independent experiments for each antagonist.

 Phα1β caused only discreet inhibition (13.55%) of hERG channel activity at 900 pM, with an IC50 > 900 pMol (Figure 1 A) In contrast, dofetilide, a known hERG antagonist used as a positive control, caused concentration-dependent blockade of the hERG channel, with a maximal inhibition of 80.6% at 10 µM and an IC_50_ of 0.1642 ± SD (Figure 1 B). 

### Cytotoxicity of Phα1β in HEK293-hERG cells

**Figure 2.**
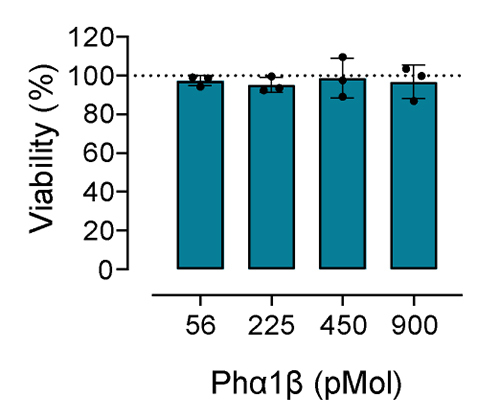
The viability of HEK293-hERG cells after incubation with Phα1β (56-900 pM) for 24 hours. Cell viability was assessed using the MTT assay. The percentage viability was calculated relative to the viability of cells incubated without toxin (control, considered as 100%). The columns represent the mean ± SD of three independent experiments, each conducted in duplicate. The filled circles indicate the values of the individual experiments. Phα1β did not alter cell viability at the concentrations tested (one-way ANOVA followed by Dunnett's test).

For the cytotoxicity assay, concentrations of 56, 225, 450 and 900 pMol of Phαβ were incubated with HEK293-hERG cells. After 24 hours incubation period, Phα1β was not cytotoxic to HEK293-hERG cells (Figure 2). 

## Discussion

For the experiments, the FLIPR Potassium assay kit (Molecular Devices) was used. This thallium influx assay provides a robust and reliable method for evaluating the ability of drugs to inhibit the hERG channel protein [28, 29, 30]. The activities of many hERG inhibitors in the thallium flux assay are consistent with those obtained in automated patch-clamp experiments [30]. The results obtained in this assay correlate with those for electrophysiology [29, 30].

The regulatory guidelines (ICH S7B) recommend inhibition of the delayed rectifier current (IKr), carried by the human hERG potassium channel-related gene (hERG) channels in cardiac cells (the hERG test), shown in this work, as a first-line test for identifying compounds that induce QT prolongation. Chemical inhibition of the human hERG potassium channel-related gene (hERG) potassium channel prolongs the QT interval, which can contribute to severe cardiotoxicity [29]. Compounds that produce TdP in humans also inhibit the rapid form of the delayed rectified potassium current IKr, encoded by the hERG gene. The adverse effects of hERG inhibition are one of the principal causes of drug attrition in clinical and preclinical development [29].

## Conclusion

Our findings indicate that even at high concentrations, Phα1β does not inhibit the functionality of hERG channel and nor affects cell viability.

## Data Availability

The raw data and the final report were stored in the CIEnP Archive (Florianópolis, Brazil) for at least five years, in accordance with POP B.07. The test item will be stored in the CIEnP archive until its validity expires or indefinitely, for at least five years (POP F.12).
